# Apraxia, pantomime and the parietal cortex

**DOI:** 10.1016/j.nicl.2014.05.017

**Published:** 2014-06-05

**Authors:** E. Niessen, G.R. Fink, P.H. Weiss

**Affiliations:** a Cognitive Neuroscience, Institute of Neuroscience & Medicine (INM-3), Research Centre Jülich, Jülich, Germany; b Department of Neurology, University Hospital Cologne, Cologne, Germany

**Keywords:** Stroke, fMRI, Lesion mapping, Motor cognition, Tools

## Abstract

Apraxia, a disorder of higher motor cognition, is a frequent and outcome-relevant sequel of left hemispheric stroke. Deficient pantomiming of object use constitutes a key symptom of apraxia and is assessed when testing for apraxia. To date the neural basis of pantomime remains controversial. We here review the literature and perform a meta-analysis of the relevant structural and functional imaging (fMRI/PET) studies.

Based on a systematic literature search, 10 structural and 12 functional imaging studies were selected.

Structural lesion studies associated pantomiming deficits with left frontal, parietal and temporal lesions. In contrast, functional imaging studies associate pantomimes with left parietal activations, with or without concurrent frontal or temporal activations. Functional imaging studies that selectively activated parietal cortex adopted the most stringent controls.

In contrast to previous suggestions, current analyses show that *both* lesion and functional studies support the notion of a left-hemispheric fronto-(temporal)-parietal network underlying pantomiming object use. Furthermore, our review demonstrates that the left parietal cortex plays a key role in pantomime-related processes. More specifically, stringently controlled fMRI-studies suggest that in addition to storing motor schemas, left parietal cortex is also involved in activating these motor schemas in the context of pantomiming object use. In addition to inherent differences between structural and functional imaging studies and consistent with the dedifferentiation hypothesis, the age difference between young healthy subjects (typically included in functional imaging studies) and elderly neurological patients (typically included in structural lesion studies) may well contribute to the finding of a more distributed representation of pantomiming within the motor-dominant left hemisphere in the elderly.

## Highlights

• Structural and functional imaging findings on tool-use pantomime converge
• A left hemispheric fronto-parietal network is involved in pantomiming tool-use
• The left (inferior) parietal cortex (IPL) plays a key role in this network
• IPL triggers the internal activation of a motor schema associated with a pantomime

## 1 Introduction

Apraxia is a disorder of higher motor cognition and a common sequel of left hemispheric stroke (Goldenberg, 2009). Apraxia significantly impacts upon rehabilitation: after discharge from the rehabilitation unit apraxic stroke patients depend more on their caregivers and return less frequently to work than patients without apraxia (Dovern et al., 2012). Frequently observed clinical symptoms of apraxia are deficits of i) imitating abstract/meaningless and symbolic/meaningful gestures, ii) pantomiming the use of objects and tools (Goldenberg et al., 2003), and iii) actual object use, in particular when complex sequential actions including multiple objects are required (Dovern et al., 2011).^1^ These deficits are assumed to represent impairments of the structural (for meaningless gestures) and the semantic (for meaningful gestures including pantomime) action processing route (Rumiati et al., 2010a) which may correspond to the dorso-dorsal and ventro-dorsal streams, respectively (Binkofski and Buxbaum, 2013). Accordingly, most studies investigating the ecological relevance of apraxia (e.g., (Hanna-Pladdy et al., 2003)) used both meaningful and meaningless items, items that tap into both the semantic and the structural processing domain (Dovern et al., 2012). To further our insights into the relationship between the two action routes and their relation to the various symptoms of apraxia is likely to result in a deeper understanding of the pathophysiology underlying apraxia. Due to high sensitivity and specificity, tests of pantomiming the use of objects and the imitation of meaningless hand gestures are considered the “gold standard” for detecting apraxic deficits related to the semantic and structural processing route. While there is consensus that the (inferior) parietal cortex is essential for imitation (Mengotti et al., 2013; Rumiati et al., 2009, 2010b), the neural basis of pantomime is debated (Frey, 2008; Goldenberg, 2009; Kroliczak and Frey, 2009; Vingerhoets, 2014). To elucidate the issue, we here perform both a review of the literature and a meta-analysis of the relevant structural and functional studies concerned with the neural basis of pantomime of object use.

When we refer to pantomime of object use, we mean the process of eliciting a meaningful, transitive movement. This can be triggered either by a name of a tool or by showing its picture. A prerequisite for pantomiming object use is the activation of the motor schema that matches the physical affordances of the object. A second important requirement for a correct pantomime of object use is the proper execution of that motor schema without the object being present. While during the actual handling of objects many motor parameters are determined by the structural properties of the object, these motor parameters have to be generated internally in the case of pantomiming object use (i.e., in the absence of the object). For example, the width of the grip holding the pretended glass (grasping component) and the distance between the hand and the mouth (transport component) during the pantomime of drinking from a glass constitute such key motor parameters. Note, however, that Laimgruber and colleagues (Laimgruber et al., 2005) demonstrated by means of kinematic analyses that pantomimes change features of movement execution: Compared to actual drinking, the width of the hand aperture was significantly reduced during pantomime of object use not only in stroke patients but also in healthy control subjects. These changes were, however, most prominent in patients with left brain damage (LBD), in whom the hand aperture was often absent during the pantomime. Taken together, the initiation and proper execution of the appropriate motor schema associated with a given object are the two main aspects of the pantomiming task, the performance of which is specifically disturbed in patients with LBD and apraxia (Goldenberg et al., 2007; Weiss et al., 2008). Accordingly, we here focus on those structural and functional imaging studies which tapped these two key processes underlying pantomiming object use. Consequently, studies in which pantomimes (shown on a video tape or produced by the experimenter) were only *imitated* were not considered, since the task of imitating a pantomime does not require the (internal) initiation (trigger) of the appropriate motor schema. In contrast, studies that used videotapes of pantomimes to test the subjects' ability to recognize or to evaluate a pantomime were included in the current analysis, since the initiation of the appropriate motor schema is a prerequisite of these tasks: In order to recognize a pantomime as ‘hammering’ or to judge whether the shown pantomime of ‘hammering’ is properly executed (i.e., the correct motor parameters are generated in the absence of the object, here: a hammer), subjects have to initiate the appropriate motor schema of hammering so that they can compare it to the pantomime shown. Likewise, studies on pantomime recognition that used videos of gestures with actual objects (Nelissen et al., 2010; Pazzaglia et al., 2008) had to be excluded, because the cognitive processes during the observation of actions with and without corresponding object are essentially different (Weiss et al., 2008). In 1982, Heilman, Rothi and Valenstein proposed a model to explain processes related to gesture execution and discrimination which actually support our current view. According to these authors, visual (when viewing objects) or linguistic (after verbal command) input is transferred to the left parietal cortex, which in turn activates premotor and motor areas for movement execution. The motor schema for a given object-related movement is supposed to be stored in the left IPL. Even though gesture (or pantomime, in our case) execution and discrimination are apparently distinguishable cognitive functions, the processes up to the activation of the appropriate motor schema are likely to be identical (see also (Goldenberg, 1999)). Heilman and colleagues support their model by reporting patient data: Whereas patients with lesions to the IPL are unable both to execute and to discriminate a gesture, patients with anterior lesions sparing the IPL exhibited deficits only in gesture execution, while gesture discrimination was preserved (Rothi et al., 1986). The authors explained this latter pattern of results by a disconnection of parietal and motor areas. Once the motor schema has been activated, the processes related to execution and recognition/discrimination of gestures obviously differ. Therefore, we would like to argue that the execution and the discrimination/recognition of a gesture both rely on the activation of the same motor schema (see also below the discussion of motor schemas for pantomiming object use and actual object use).

After clarifying the motor cognitive processes underlying pantomime of object use, we now turn to the recent debate about the neural basis of pantomiming object use. As stated above, deficits in pantomiming the use of objects and tools are most frequently observed in patients with left brain damage. Traditionally, the left parietal lobe has been considered an important region for pantomiming object use (Rothi et al., 1985, 1986). Consistently, early functional imaging studies of pantomiming tool use following verbal command observed activations within the left parietal lobe (i.e. (Moll et al., 2000; Choi et al., 2001)). Recently, however, it has been argued that these functional imaging data obtained from healthy subjects are at odds with findings in patients (Bohlhalter et al., 2011; Fridman et al., 2006; Goldenberg, 2009; Kroliczak and Frey, 2009). The importance of the parietal cortex for pantomime of object use was questioned based upon the observation that pantomime of object use performance was similar for patients with and without left parietal lesions (Goldenberg et al., 2003). Furthermore, in a lesion study of aphasic patients with left hemispheric stroke Goldenberg and colleagues showed that especially left inferior frontal lesions resulted in deficient pantomime of object use, whereas left parietal lesions did not significantly impair pantomime performance (Goldenberg et al., 2007). Studies using neuromodulation (Bohlhalter et al., 2011) and functional imaging (Bohlhalter et al., 2009; Fridman et al., 2006) further supported the importance of the frontal (and premotor) cortex for transitive actions (and thus pantomiming). On the other hand, there is growing evidence that the parietal cortex integrates representations for complex tool-use skills (e.g., conceptual knowledge about objects and their functional use) that are computed in a distributed network of regions (Frey, 2008; Vingerhoets, 2014). Therefore, both the specific function of the parietal cortex in pantomiming the use of objects and the contribution of the regions participating in the pantomiming network need to be clarified using a meta-analytic approach to resolve the apparent discrepancies between the results of functional neuroimaging studies (in young healthy subjects) and the findings of structural lesion studies (in elderly neurological patients). This approach will also further our understanding of the pathophysiology of apraxia.

## 2 Methods

In order to assemble all studies investigating the neural correlates of pantomime of object use, we used the search engine ‘PubMed’. First, we used the terms ‘pantomime’ AND ‘apraxia’ AND ‘lesion’ to identify structural lesion studies. Secondly, we selected functional imaging studies related to the terms: ‘pantomime’ AND ‘tool use’ AND ‘imaging’. Note that to date no single functional imaging study exists which examines pantomimes of object use in stroke patients. For further inspection, the reference lists of the appearing studies were searched for relevant, yet undetected papers. Overall, we initially identified 66 relevant studies and then restricted our selection to studies that applied pantomiming tests according to our definition, i.e., conceptualizing pantomime of object use as the process of eliciting a meaningful, transitive movement triggered either by the name of the tool or by a picture of the tool.

Furthermore, single case studies and review papers were excluded. As the scope of this review was to evaluate the involvement of specific brain regions in pantomiming object use, only those studies were included in our analysis that associated deficits in pantomiming object use with specific lesion sites, or, for functional imaging studies in healthy subjects, related the pantomime task to circumscribed activation clusters. For example, Fazio et al. (2009) applied a neuropsychological test battery consisting of tests of language comprehension, imitation and pantomime, to six patients with a stroke in the territory of the left middle cerebral artery (MCA). Unfortunately, though, test performance was reported only for the overall test battery (i.e., no performance parameters were given for the individual tests). Therefore, that study could not provide any conclusive information about the association between lesion sites in the examined patient sample and pantomime of object use performance. Thus, the study by Fazio and colleagues was not included in further analyses.

In addition, studies were excluded if pantomime deficits were reported for a heterogeneous patient group. Goldenberg et al. (2003) investigated pantomiming actions in a group of 52 stroke patients and reported significant deficits after LBD. However, within the group of LBD patients (n = 40), individual lesions were observed in the frontal, parietal, occipital, and temporal cortex. The severity of pantomiming deficits was documented for the entire LBD group only, i.e., no information about the pantomime performance of individual patients was provided. Therefore, the reported data did not allow to associate pantomime deficits with specific lesion sites. Accordingly, that study was not included in our analyses.

For the following reasons, we restricted our analyses on left hemisphere regions only: First of all, the left hemisphere is known to be motor dominant in right-handers who constitute about 90% of the population (Forrester et al., 2013). In right-handers, deficient pantomime of object use, and thus apraxia, predominantly arises after LBD (Goldenberg et al., 2003; Heilman and Rothi, 1993). Consistent with this observation, functional imaging studies have repeatedly shown that pantomime of object use activates a left-lateralized network, even when the pantomimes are executed with both hands (Ohgami et al., 2004). Accordingly, data on pantomime deficits in patients with right hemispheric brain damage (RBD) were not considered in the current review although these warrant further investigation (Weiss et al., 2006). For consistency, we also refrain from further analyzing (and discussing) right-hemispheric activations reported in some functional imaging studies of pantomiming object use (e.g., Johnson-Frey et al., 2005).

**Fig. 1:**
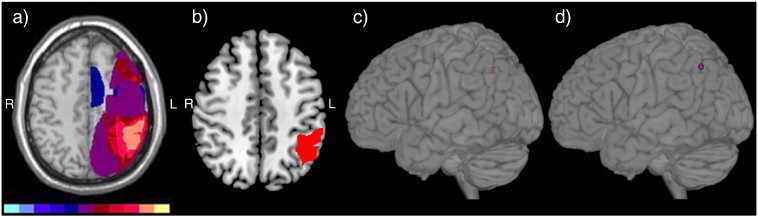
Explanation of the procedure used to depict the anatomical location of lesion findings in the rendered template brain. a) Original illustration by Buxbaum et al. (2005); orange color indicate the maximum lesion overlap. b) The maximum lesion overlap was projected onto the corresponding slice (z = 46) of the standard template brain provided by MRIcron. If the z-coordinate was provided, we used the respective axial slice of the standard template brain; if not we selected the appropriate slice by comparing macro-anatomical landmarks (as in this case). Please note that Buxbaum et al. (2005) used a template (ch2) of the software MRIcro, whereas we used a template (ch2better.nii.gz) of the more recent software MRIcron. Therefore, small differences in the macro-anatomy between the original figure and the current template brain are inevitable. Furthermore, for lesion mapping, the neuro-radiological/neurological convention is used, i.e., the left hemisphere is shown on the right side. c) Then, the standard template brain including the lesion map with the maximum lesion overlap was rendered. Accordingly, the maximum lesion overlap was visible on the rendered surface of the template brain. d) Finally, a symbol was used to indicate the location of the visible portion of the maximum lesion overlap corresponding to the lesion location in the original illustration/study.

It is important to note that the structural studies which investigated the neural correlates of pantomiming in patients used different lesion mapping methods. While early lesion studies adopted descriptive methods (e.g. (Buxbaum et al., 2005)), quantitative, statistical lesion mapping was introduced in more recent studies (e.g. (Weiss et al., 2008)). The most commonly used descriptive methods are lesion overlay and subtraction plots (Goldenberg and Karnath, 2006). Lesion overlay plots show the extent to which a given brain region is affected by the lesions of a patient group investigated. Within the overlay plot, those regions that are affected more frequently than others are color-coded to illustrate the number of patients suffering from a lesion in a specific location (for example, see Fig. 1a of (Goldenberg and Karnath, 2006)). Typically, the brain region affected by the largest number of patients is associated with the behavioral deficit. Descriptive subtraction plots are used for the comparison of patient groups with and without a given behavioral deficit (most often defined by a cut-off score for impairment). These plots are generated by subtracting the lesion patterns of the patient groups: brain regions, which are more frequently affected in the group suffering from the impairment under study, are then color coded. Thus far, however, no statistical tests are applied; rather, the lesion distribution is qualitatively described (e.g., by the percentage of overlap differences; for example, see Fig. 1b of (Goldenberg and Karnath, 2006)). In contrast, advanced lesion methods apply specific statistical tests (Rorden and Brett, 2000; Rorden et al., 2007). For group comparisons, the Liebermeister test (e.g., (Vossel et al., 2012)) was found to be more sensitive than the formerly used chi-square test (e.g., (Weiss et al., 2008)). These tests when properly applied reveal the brain regions that are *significantly* more affected in the patient group suffering from a given cognitive impairment when compared to a patient group unaffected by that cognitive impairment. This analysis obviously depends on the criteria used to define the two patient groups (usually a cut-off score in a given cognitive test). Voxel-based lesion symptom mapping (VLSM; (Bates et al., 2003; Kimberg et al., 2007)) circumvents this problem: VLSM assesses the statistical relationship between behavioral measures (here: the performance of neurological patients in a cognitive test) and the structural integrity of brain regions on a voxel-by-voxel basis. Thus, this method does not depend on the a-priori division into two groups (e.g., by a cut-off-score), but rather uses the power of the whole patient sample by evaluating for each and every voxel whether the mean test performance of the patient group, in which that voxel is affected by the lesion, is statistically different from the mean test performance of those patients, whose lesions do not comprise the voxel under investigation. Therefore, the key advantages of VLSM are that this method (i) relies on continuous behavioral data, and (ii) is independent of an a-priori categorization of the patient. Medina and colleagues (Medina et al., 2010) recently showed that the statistical inference in VLSM analyses of (small) patient populations should be based on the parametric *t*-test with permutation derived correction (Kimberg et al., 2007) rather than on the Burner–Munzel-Test (Rorden et al., 2007).

As apparent discrepancies between structural and functional studies have been discussed previously (Frey, 2008; Goldenberg, 2009), we depict the location of the reported lesion sites and activation clusters in separate figures. The graphical illustration of common activation sites or lesion sites was accomplished by using anatomical landmarks or MNI-coordinates reporting the location of the main peak of activity (maximally activated voxel) within separate activation cluster in a given study. If coordinates were reported in Talairach space, we transformed them into MNI-coordinates by using a standardized tal2mni algorithm (Matthew Brett, http://eeg.sourceforge.net/doc_m2html/bioelectromagnetism/mni2tal_matrix.html). These landmarks or coordinates were then used to schematically display the location of the pantomime-related activations or lesions on the rendered template brain provided by MRIcron (Rorden et al., 2007). To ensure that the anatomical labels were consistent across all studies included in this review, we assigned anatomical labels to activation peaks based on their MNI-coordinates by applying the freely available SPM Anatomy Toolbox (Eickhoff et al., 2005). If no graphical display but rather a detailed anatomical description of a lesion was provided (e.g., an enumeration of Brodmann areas (BA) affected by the lesion (Buxbaum et al., 2003)), we then checked these tables for common lesion sites (i.e., BAs affected in all patients) and used these to localize symbols in our figures. For example, Buxbaum et al. (2003) report in Table 2 (page 1098) that all their apraxic patients with pantomiming deficits suffered from lesions affecting both BA 39 and BA 40 (with one exception, in whom only BA 40 was affected). Therefore, the symbol for the study of Buxbaum et al. (2003) was placed in the inferior parietal cortex at the border of BA 39 (supramarginal gyrus) and BA 40 (angular gyrus). The same procedure was also used for the other study of Buxbaum et al. (2005): Compared to the control group and non-apraxic patients, their apraxic patients were severely impaired in the task “gesture to sight of objects” (i.e., pantomiming the use of (visually presented) objects; see their Table 3 on page 925). Therefore, we extracted the common lesion site within the apraxic patient group with the same procedure as described above based on their Table 1 on page 920 (Buxbaum et al., 2005). If, as in this example, symbols were positioned based on rough anatomical descriptions, then these symbols were shaded to distinguish them from symbols that were positioned based on exact coordinates or lesion maps (continuous coloring). The methodology used to convert the lesion findings of structural studies in which only figures with lesion overlaps without precise anatomical descriptions were presented to proper locations on the rendered template brain is depicted in Fig. 1. Note that some studies reported multiple regions. In this case, the same symbol was used to denote the different lesion sites or activation clusters reported in a given study.

**Fig. 2:**
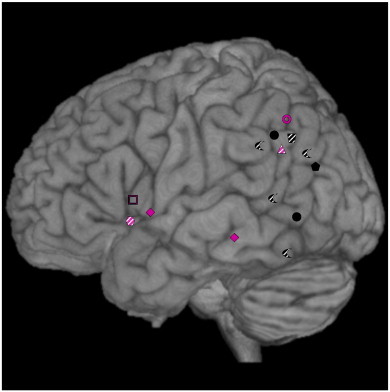
Lesion sites associated with deficient pantomime in stroke patients as reported by structural imaging studies. The color of the symbols differentiate two behavioral tasks: lesion sites found in studies involving the execution of pantomimes are depicted by purple symbols, while those from studies examining the recognition of pantomime are depicted by black symbols. The transparent purple hexagon depicts a lesion located in the insula (Hermsdörfer et al., 2013), which was projected to the lateral surface of the cortex. Shaded symbols are positioned based on rough anatomical descriptions. The different symbols indicate the corresponding studies:  = Varney and Damasio, 1987;  = Halsband et al., 2001; ▲ = Buxbaum et al., 2003;  = Buxbaum et al., 2005;  = Goldenberg et al., 2007;  = Weiss et al., 2008; ● = Kalenine et al., 2010; ♦ = Manuel et al., 2013;  = Hermsdörfer et al., 2013. Note that one lesion location in the basal ganglia is not shown in the figure (Varney and Damasio, 1987).

**Fig. 3:**
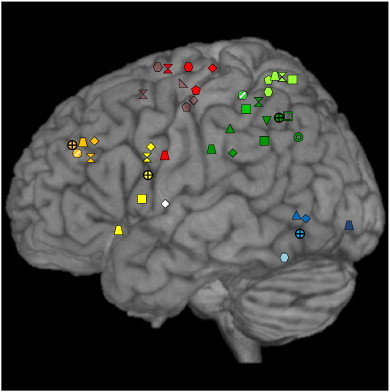
Cortical activation sites associated pantomime in healthy subjects as reported by functional imaging studies. Color-coding is used to group the corresponding activation clusters by anatomical region (orange = middle frontal gyrus (MFG); yellow = inferior frontal gyrus (IFG); green = intra-parietal sulcus (IPS); dark green = inferior parietal lobe (IPL); light green = superior parietal lobe (SPL); blue = medial temporal lobe; light blue = inferior temporal lobe; dark blue = inferior occipital gyrus (IOG); red = lateral precentral gyrus; transparent red = medial precentral gyrus; white = insular cortex). Shaded symbols are positioned based on rough anatomical descriptions. The different forms identify associated studies: ● = Moll et al., 2000;  = Choi et al., 2001; ♦ = Rumiati et al., 2004;  = Ohgami et al., 2004; ▲ = Johnson-Frey et al., 2005; ■ = Fridman et al., 2006; ▼ = Imazu et al., 2007;  = Hermsdörfer et al., 2007;  = Bohlhalter et al., 2009;  = Kroliczak and Frey, 2009;  = Vingerhoets et al., 2011;  = Vingerhoets et al., 2012. The four crosshair symbols () show the results of the ES-SDM analysis. Note that in addition to the displayed cortical activation sites, three studies reported also non-cortical activation sites (not shown): in the putamen (Choi et al., 2001; Rumiati et al., 2004) and in the cerebellar vermis (Rumiati et al., 2004; Vingerhoets et al., 2012).

In addition to the symbol coding, a color coding was applied. The color-coding system served different purposes in the two figures: Whereas different colors in the structural lesion graph (Fig. 2) depict different types of cognitive tasks (i.e., purple means execution of pantomimes; black means assessment of pantomimes), the color-coding in the functional imaging map (Fig. 3) indicates different anatomical regions in which the activation peaks were clustered (e.g., the color green denotes the inferior parietal cortex). Furthermore, to facilitate comparisons, activation peaks originating from cortical areas located on the medial surface (e.g., medial precentral gyrus/medial Brodmann area 6) or in sulci (e.g., IPS) as well as insular activations were projected to the lateral surface of the rendered template brain in Figs. 2 and 3.

In addition to the above specified descriptive approach, we conducted a quantitative meta-analysis (effect-size signed differential mapping (ES-SDM); (Radua and Mataix-Cols, 2012)) of all included functional imaging studies that reported coordinates of main activation peaks to quantitatively identify the anatomical regions that were associated with object-related pantomimes across studies. The ES-SDM method computes a quantitative meta-analysis of functional imaging results by not only taking into account the coordinates and effect-sizes of the activation clusters' main peaks, but also the number of participants (i.e., the underlying power of the reported results in a given study). Since ES-SDM works with Talairach coordinates, reported MNI-coordinates were converted for ES-SDM and the ES-SDM-results were again converted to the MNI-system using the tal2mni algorithm. Finally, the anatomical labels were derived from the SPM Anatomy Toolbox (Eickhoff et al., 2005).

## 3 Results

In total, 22 studies (10 structural lesion studies and 12 functional imaging studies) were selected according to the above defined criteria and further evaluated. Table 1 (structural lesion studies) and Table 2 (functional imaging studies) provide further details of the studies that entered the final analysis (e.g., experimental design, study population etc.). For functional neuroimaging studies, the control condition is listed, as well as the sensory modality, in which the stimuli were presented, and the stimuli (object name, photo of object or written command of specific movement) which triggered the pantomime.

### 3.1 Structural lesion studies of the neural basis of pantomiming

Ten structural lesion studies were analyzed (see Table 1). Of those, six examined executed pantomimes, while four explored the recognition of pantomimes. Patients with left-hemispheric lesions were consistently asked to use their ipsilesional, left hand during task performance. Furthermore, the study by Manuel et al. (2013) constitutes the only retrospective study included in this review. Manuel and colleagues included stroke patients, who had undergone pantomiming tests in the past 4 years. With this retrospective design, Manuel and coworkers managed to recruit a large sample of patients (n = 150).

The resulting overview plot of structural lesion studies is provided in Fig. 2. With respect to the macro-anatomical location of the main lesion sites, lesions of the middle temporal gyrus (MTG) were associated with pantomiming deficits in two studies (Kalenine et al., 2010; Manuel et al., 2013), and one study reported lesion locations in the superior temporal gyrus (STG; (Varney and Damasio, 1987)). The inferior parietal lobe (IPL) was associated with an impaired pantomiming performance in six studies (Buxbaum et al., 2003, 2005; Halsband et al., 2001; Kalenine et al., 2010; Varney and Damasio, 1987; Weiss et al., 2008). In one study, lesions affecting the inferior frontal gyrus (IFG) were found to impair the execution of pantomimes (purple open square in Fig. 2 (Goldenberg et al., 2007)). Most likely due to the large sample size (n = 150) and hence the enhanced statistical power combined with a sensitive analysis (VLSM), Manuel et al. (2013) additionally identified a frontal lesion site, i.e., the pars operculum of the IFG (purple diamond in Fig. 2). One study related lesions of the insula to pantomiming deficits (purple hexagon in Fig. 2 (Hermsdörfer et al., 2013)). Varney and Damasio (1987) additionally reported lesions in the basal ganglia and in the fusiform gyrus which were associated with deficits in pantomime recognition.

In contrast, Dovern et al. (2011) could not find a clear association between a specific lesion site and pantomime of object use deficits in their VLSM-analysis of 43 left-hemisphere stroke patients. The authors discussed whether their sample was too small to reveal a significant lesion–symptom association for pantomime of object use, although their VLSM-analyses revealed the inferior parietal cortex as the critical lesion site for hand gesture imitation deficits (and thus replicated previous lesion studies on imitation (Goldenberg and Karnath, 2006)). The most parsimonious explanation of their findings is that pantomimes of object use can be affected by lesions to a network of areas rather than a single lesion site only.

In summary, of 14 reported lesion locations associated with pantomime deficits 7 were located in the parietal cortex (50%), 2 in the frontal cortex (14%), 3 in posterior temporal regions (22%), and one in the insula and fusiform gyrus (each 7%). One study did not single out a specific lesion correlate for pantomiming deficits (Dovern et al., 2011).

### 3.2 Functional imaging studies on the neural basis of pantomiming

According to the above reported selection criteria, 12 functional imaging studies were included in the current review that were conducted with healthy subjects only. Predominantly, these studies opted for fMRI as imaging method; one study was conducted with PET (see Table 2). With respect to the hand used for pantomiming object use, in 10 functional imaging studies pantomimes were performed with the right hand only or activation clusters for right hand pantomimes were separately reported from those for left hand pantomimes (4 of the 10 studies also included left hand performance). The remaining two studies (Hermsdörfer et al., 2007; Moll et al., 2000) reported the common activation clusters related to pantomimes executed with the left and the right hand in successive blocks. Fig. 3 presents the overview plot of the functional imaging results. Note that we color-coded the activation sites according to the macro-anatomical brain regions in which they were located.

Pantomiming movements elicited posterior temporal activations in three studies: one in the inferior temporal gyrus (ITG; light blue) (Choi et al., 2001) and two in the MTG (blue) (Johnson-Frey et al., 2005; Rumiati et al., 2004). One activation was found in the inferior occipital gyrus (IOG; dark blue) (Vingerhoets et al., 2012). Within the middle frontal gyrus (MFG; orange), four activation clusters were associated with the execution of pantomimes (Moll et al., 2000; Ohgami et al., 2004; Rumiati et al., 2004; Vingerhoets et al., 2012). The study by Ohgami et al. (2004) reported additional pantomime-related activations in the IFG (yellow), as did Rumiati et al. (2004), Fridman et al. (2006) and Vingerhoets et al. (2012). Rumiati et al. (2004) also related activity of the insular cortex (white) to pantomimes.

However, the majority of activations were located in the parietal lobe. The superior parietal lobe (SPL; light green) was reported to be activated during pantomiming in five studies (Bohlhalter et al., 2009; Choi et al., 2001; Fridman et al., 2006; Ohgami et al., 2004; Vingerhoets et al., 2012). Furthermore, three studies found pantomime-related activations in the IPS (green (Fridman et al., 2006; Hermsdörfer et al., 2007; Moll et al., 2000)). Note that the green open square (Hermsdörfer et al., 2007) also denotes an IPS activation. Finally, seven functional imaging studies revealed significant IPL activations (dark green) during pantomiming (Fridman et al., 2006; Imazu et al., 2007; Johnson-Frey et al., 2005; Ohgami et al., 2004; Rumiati et al., 2004; Vingerhoets et al., 2011, 2012).

Furthermore, five studies reported activations associated with pantomime performance in the lateral BA 6 (Bohlhalter et al., 2009; Choi et al., 2001; Ohgami et al., 2004; Rumiati et al., 2004; Vingerhoets et al., 2012) as defined by the SPM Anatomy Toolbox and five studies observed pantomime-related activations in the medial BA 6 (transparent red) (Bohlhalter et al., 2009; Choi et al., 2001; Kroliczak and Frey, 2009; Ohgami et al., 2004; Rumiati et al., 2004).

Further non-cortical activations were reported in the cerebellum (Choi et al., 2001; Vingerhoets et al., 2012) and in the putamen (Choi et al., 2001; Rumiati et al., 2004).

All but one (Kroliczak and Frey, 2009) of the studies that found frontal, temporal, precentral (corresponding to BA 6), occipital or insular activations associated with pantomime concurrently also reported parietal activations. On the other hand, only three studies selectively elicited activations within the parietal lobe (Fridman et al., 2006; Hermsdörfer et al., 2007; Vingerhoets et al., 2011).

In line with this qualitative evaluation, the ES-SDM analysis revealed a similar pattern of results (see Table 3). According to this quantitative meta-analysis, pantomiming object use is significantly related to activity in the left IPL (greatest cluster of overlapping voxels), as well as activity in the left IFG, the left MFG and the left inferior temporal gyrus (see Fig. 3, symbols with crosshairs).

## 4 Discussion

The current review investigated the neural correlates of pantomime of object use as indicated by structural lesion and functional imaging studies. Recently, it was argued that the results from lesion studies are inconsistent with the findings obtained from imaging studies (Frey, 2008; Fridman et al., 2006; Goldenberg, 2009). The results of our comprehensive analysis and the ES-SDM approach reconcile the findings from structural and functional imaging studies since they provide converging evidence for an important role of the parietal cortex as a key node within a left-hemispheric network subserving pantomiming: In addition to the predominant involvement of the parietal lobe in functional imaging studies of pantomime of object use (nearly all functional imaging studies revealed activation peaks located in the parietal lobe), a similar trend could be observed for the lesion studies, where six studies (i.e., 60% of the evaluated structural lesion studies) showed that lesions affecting the parietal lobe were associated with pantomime deficits. Thus, structural and functional imaging studies converge regarding the importance of the parietal cortex for pantomiming the use of objects. Nevertheless, the analysis also clearly suggests that functional imaging of healthy subjects and lesion analyses in patients provide differential information. In the following, reasons for these discrepancies will be discussed. Furthermore, based on the analysis of the experimental paradigms we will put forward a hypothesis about the specific motor cognitive role of parietal cortex in pantomiming.

Importantly, all but one of the functional imaging studies reporting frontal, insular, precentral/premotor or temporal activations during pantomime revealed concurrent activations in the left parietal cortex. This suggests that pantomiming is supported by a left-hemispheric network in which the parietal cortex plays a key role. Results of the ES-SDM analysis supported this notion, suggesting that the left hemispheric network subserving object use pantomimes is composed of the inferior parietal lobe, the inferior frontal gyrus, and to a lesser degree of middle frontal and inferior temporal regions. The extensive cluster of significant voxels in the IPL underlines its special importance within the pantomime network (i.e., the number of voxels amounted to more than three-times (2570) than those in the inferior frontal gyrus (843 voxels), see Table 3). To further decode the critical function of the left parietal lobe in the context of pantomiming of object use, we also examined which features of the experimental paradigm and procedures were specific to the three studies that selectively activated the parietal cortex during pantomimes (Hermsdörfer et al., 2007; Imazu et al., 2007; Vingerhoets et al., 2011): One characteristic, which separates the three studies from the other functional imaging studies on pantomime, is the control task employed. Two of these studies, i.e., Hermsdörfer et al. (2007) and Imazu et al. (2007), compared brain activity related to pantomimes with activations associated with the actual use of the same objects.

In contrast, other functional imaging studies used meaningless movements as control (Johnson-Frey et al., 2005; Moll et al., 2000). In the study by Moll et al. (2000), participants learned a multistage sequence of movements and were asked to repeat this sequence in a step-by-step fashion during the control condition. In contrast, Johnson-Frey et al. (2005) instructed their participants to deliberately execute a random and meaningless movement, trying to avoid repetitions. These control conditions aimed at achieving a movement complexity comparable to that of pantomimes. However, one of the requirements for the performance of a correct pantomime is the proper execution of the internally triggered motor schema (i.e., the proper implementation of the specific movement kinematics and motor parameters). This specific aspect of a pantomime of object use is not adequately controlled for by relatively simple, meaningless movements. Even though in the case of Moll et al. (2000) the sequence of movements was learned just prior to the fMRI measurement, it cannot be assumed that the representation of the motor schema underlying the multistage sequence of meaningless control movements was comparable to that of motor schemas representing overlearned, object-related and meaningful movements. Furthermore, since a proper pantomime of object use should draw upon identical or similar motor parameters as the actual object use, using deliberately chosen movements for control most likely revealed the motor network common to pantomiming and using objects. This ‘object use’ network is known to comprise of widespread left-hemisphere regions including not only parietal cortex, but also frontal and temporal cortices (Binkofski et al., 1999; Johnson-Frey and Grafton, 2003).

The two studies adopting actual object use as control for pantomiming did not only properly control for motor parameters, but also tried to assimilate the subjects' intentions in these two conditions (here: demonstrating the use of objects). The study by Vingerhoets et al. (2010) for instance showed that different brain regions are involved when subjects observe a grasping movement that is intended to use an object, or when they observe a grasping movement which aims at displacing the object. More specifically, they reported that several portions of the IPS (anterior, middle and caudal) were active during an intention discrimination task, i.e., during the observation of two movements that only differed with respect to the intention of the subsequent movement (using versus displacing the object). Taken together, comparing pantomiming movements with actual object use ensures that the parameters and the intentions of the compared movements resemble each other as close as possible.

Furthermore, it is assumed that the motor schema, which has to be activated prior to the execution of a transitive movement, is identical for pantomimed and actual object use. According to the model by Heilman et al. (1982) described above, the motor schema is stored in the IPL. Also more recent work by Rumiati et al. (2004) identifies the IPL as the storage site of the motor schema in the context of object-related pantomimes. Supporting evidence was reported by Vingerhoets et al. (2012), who associate the IPL with storage of motor schemas related to familiar objects.

The remaining crucial difference between the two tasks (pantomiming versus actual use) lies in the way the appropriate motor schema for a given object is triggered. In the pantomime condition, the motor schema has to be triggered internally, whereas during real object use, the physical properties of the object trigger the appropriate motor schema. This suggests that this internal triggering of the motor schema in response to the picture of an object or the processing of its name is characteristic for pantomiming object use. According to the current review this specific motor cognitive component of the pantomime task selectively activates the left inferior parietal cortex (Hermsdörfer et al. (2007) and Imazu et al. (2007)). Unfortunately, to date a more detailed characterization of the critical locus within the inferior parietal cortex that supports this specific cognitive function for pantomiming cannot be provided, since none of the reviewed studies specifically aimed at a more precise anatomical localization (cf., however, (Weiss et al., 2013)).

It could be argued that the IPL does not only host the mechanism for triggering the motor schema, but is also the site of motor schema storage. In their PET-study (Rumiati et al., 2004), Rumiati and colleagues compared pantomiming object use after visual presentation of an object (IO) with imitating a pantomime presented as a video (IA). When both pantomiming conditions were compared to naming (the object or the action), the inferior parietal cortex (BA 40) was found to be activated (their Table 1, our Table 2). A parsimonious interpretation of this finding is that the motor schema is stored in the IPL. Interestingly, their interaction analysis (their Fig. 1) revealed two subareas within the IPL which were either related to both pantomime conditions (ventral IPL area (z = + 34), close to the IPL cluster found for the main effect of pantomiming (versus naming), z = + 29) or specifically to the condition IO (dorsal IPL area, z = + 48). Thus, the latter, more dorsally located area was specifically activated when the motor schema was triggered by the visual presentation of an object (IO), but not if the same motor schema was used for imitation (IA). We interpret these findings as follows: the ventral IPL area stores the motor schema, while the more dorsally located IPL area hosts the mechanism triggering the motor schema's execution in the context of pantomime.

Consistent with this notion, the two fMRI-studies that revealed a specific activation of the parietal cortex for pantomiming object use (Hermsdörfer et al., 2007; Imazu et al., 2007) by comparing pantomiming the use of objects with actual object use also found dorsally located parietal activation peaks (Hermsdörfer et al.: z = + 48, Imazu et al.: z = + 45). It could well be argued that the parietal activation related to the storage of the motor schema was canceled out in these studies (as both pantomiming object use and actual object use make use of the (same) motor schema) and that what remained was the activation related to the differential trigger mechanism, i.e., in case of pantomime the motor schema is internally triggered, in case of actual object use the motor schema is externally triggered by the physical properties of the object.

This notion is supported by a lesion study of Buxbaum et al. (2003). Authors observed that apraxic patients, when shown prehensile objects, produced deficient hand postures (for using these objects). Moreover, apraxic patients were impaired when asked to select, among four possibilities, the appropriate hand posture to manipulate *familiar* objects. However, the same group of apraxic patients showed a normal performance when selecting correct hand postures for *novel* objects. Note that for the latter task patients did not produce but evaluated hand postures. This set of findings suggests that the configuration of movement parameters in response to the structure of a (novel) object was intact (but see also (Sirigu et al., 1995; Goldenberg and Hagmann, 1998)), while the appropriate motor schema for familiar objects could not be activated. Consistent with imaging results from Hermsdörfer et al. (2007) and Imazu et al. (2007), the highest lesion overlap of the apraxic patients was found in the left IPL (Buxbaum et al., 2003).

The third functional imaging study that reported selective activation of parietal cortex (Vingerhoets et al., 2011) compared pantomiming the use of familiar versus unfamiliar/novel objects. A specific motor schema for a given action can only be accessed if knowledge about the manipulation of the object is already present. Therefore, unfamiliar objects cannot activate a specific pre-existing motor schema, but rather lead to the generation of a new motor schema. Thus, the comparison of pantomimes for familiar versus unfamiliar objects should reveal those brain regions that are involved in the activation of a pre-existing motor schema for familiar objects (i.e., the inferior parietal cortex) (Heilman et al., 1982; Vingerhoets et al., 2012). Therefore, the IPL activation found by Vingerhoets et al. (2011) is rather linked to the storage of motor schemas that exist for familiar objects only and are activated by pantomiming the use of familiar objects. This is in line with results from Bohlhalter et al. (2009) who contrasted the execution of transitive pantomimes with planning of the same movements. Since for both conditions the processes up to the activation of motor schemas are similar, the left IPL was possibly activated in both conditions and therefore not visible after contrasting them.

As evident from Fig. 3, the number of activations in the parietal lobe (n = 15) clearly exceeds the number of activations in the frontal (n = 8 when excluding medial and lateral BA6 activation), temporal (n = 3), occipital (n = 1) and insular (n = 1) lobe, indicating a more frequent involvement of parietal regions in pantomime. However, in those imaging studies reporting multiple activation sites, parietal regions also reached higher t- or z-scores compared to the co-activated frontal or temporal areas. Johnson-Frey et al. (2005) for instance reported a t-score of 8.13 for the pantomime-related activation in the left supramarginal gyrus (SMG; as part of the IPL), but only a t-score of 4.66 for the activation cluster in the posterior temporal lobe. Interestingly, all 6 temporal sites related to pantomime of object use (3 lesion sites (Kalenine et al., 2010; Manuel et al., 2013; Varney and Damasio, 1987)) and 3 activation sites (Choi et al., 2001; Johnson-Frey et al., 2005; Rumiati et al., 2004) lie within the posterior part of the temporal cortex (see Figs. 2 and 3). This finding is consistent with the notion that the posterior temporal cortex subserves the identification of objects in the context of action (Johnson-Frey, 2004; Lewis, 2006).

Both structural and functional imaging data point to an additional involvement of the frontal cortex in pantomiming the use of objects. As is evident from the structural lesion data, all studies that associated pantomime deficits with frontal lesions investigated the *execution* of pantomimes. In contrast, an association of pantomime deficits with lesions in the parietal and temporal lobe were reported in studies which examined the recognition/assessment of pantomimes. Therefore, lesion studies suggest that the frontal cortex is involved specifically in the execution of pantomimes.

Functional imaging studies of pantomime of object use also revealed activations in medial and lateral precentral areas (BA 6), i.e., the supplementary motor area (SMA) and the premotor cortex. Based on the above arguments, we propose that these regions are not *specifically* involved in pantomiming, but rather reflect general motor functions related to the complex task of pantomiming the use of objects. Activations of premotor regions in functional imaging studies are frequently observed and typically associated with the execution of object-related gestures per se (e.g., (Ohgami et al., 2004); see also the above discussion about actually using objects as a control task for pantomiming the use of objects). Wheaton et al. (2005) conducted an EEG study to record the temporal activation pattern during pantomime. According to their findings, neural activation during the preparation of pantomimes starts in the parietal lobe and spreads to premotor areas during the execution phase of the movement. These results also favor a specific role of the parietal cortex in pantomiming. In fact, the early parietal activation as measured by the time-sensitive EEG-method fits well with the notion proposed here that the parietal lobe subserves the internal selection and triggering of the appropriate motor schema. Wheaton et al. (2005) further suggest a general involvement of premotor areas during movement execution (both in the context of pantomiming and actual object use). Again, this is consistent with the results of the lesion studies: while parietal and temporal lesions led to deficits in assessing and executing pantomimes, frontal lesions led to deficits in executing pantomimes only (Fig. 2).

We want to stress that there is only one functional imaging study that did not report parietal activations associated with pantomime of tool use (Kroliczak and Frey, 2009). The authors compared the execution of transitive pantomimes with the execution of intransitive pantomimes (e.g., waving goodbye). This control condition is highly suitable since it is very similar with regard to movement intention and the way a pre-existing motor schema is triggered. Consistently, the subtractive analysis did not reveal parietal cortex activations. However, when inspecting the results more closely it is evident that although there is no main peak of activity within parietal cortex, there are still three sub-maxima located in the superior and inferior parietal cortex indicating slightly greater recruitment of the IPL during tool use pantomimes. The main activation peak in the precentral gyrus during transitive versus intransitive movements could be due to differences in the precision needed for the exact execution of tool-related movements. Alternatively, one could argue that differences in the direction of the movement (distal versus proximal) led to greater demands on the motor cortex during the execution of tool use pantomimes. Please also note that other contrasts reported by Kroliczak and Frey, for instance the comparison of transitive pantomimes and a linguistic control task, did reveal parietal activations.

The hand used for the execution of pantomimes was inconsistent across the included 12 functional imaging studies. For the majority of the functional imaging studies (n = 10) participants were asked to use their right, i.e., dominant hand for the execution of pantomiming movements. Two studies required their subjects to use their right and left hands in successive blocks (Hermsdörfer et al., 2007; Moll et al., 2000). Using the right hand for executing pantomimes is of course the more naturalistic approach for the right-handed subjects studied. However, Moll et al. (2000) and Hermsdörfer et al. (2007) observed the same left-lateralization of the pantomime network for tool-use pantomimes executed with the right or left hand as did studies using the right hand only (i.e. (Choi et al., 2001)). Thus, it seems that the current findings can be generalized although the reported results of two of the 12 functional imaging studies did also include activations related to the left hand: the described activation pattern seems to be related to pantomiming of object use independent of the hand used during the task. Nevertheless, the influence of handedness and responding hand on the lateralization of brain activity during pantomiming merits further investigation (cf. (Kroliczak and Frey, 2009)).

In addition to the described age difference between the populations investigated in structural lesion versus functional imaging studies, also other differences inherent to the two methods could lead to different or even discrepant findings. With respect to studies focusing on the execution of pantomimes, it should be kept in mind that in functional imaging studies participants are very restricted in their mobility and are therefore often instructed to execute the movements between their waist and chest (Hermsdörfer et al., 2007). Furthermore, the supine position during functional imaging studies requires a spatial transformation of the pre-existing motor schema to match the current reference frame (Goldenberg et al., 2007), a process that is supposed to rely on the left superior parietal lobe (SPL (Felician et al., 2004)). In contrast, for structural lesion studies neurological patients are tested in a sitting position which closely resembles the typical situation in which the objects are used during activities of daily living (drinking glass, coffee cup, cutlery, pen, etc.). Thus, there is no need for patients to adjust their motor schemas used for pantomiming to non-typical body positions.

Moreover, the demands on timing of the pantomime actions are inherently different between functional imaging and structural lesion studies. The experimental design of functional imaging studies often requires that the participants (repeatedly) execute the pantomime within a defined time window of several seconds (Fridman et al., 2006; Vingerhoets et al., 2011). In structural lesion studies on the other hand, patients usually execute the requested pantomime once only and in a self-paced, natural way. However, despite these methodological differences, we would like to stress that results from structural lesion and functional imaging studies may converge, as was nicely demonstrated by Saygin (2007).

Apart from the above mentioned differences between functional imaging and structural lesion studies, another putative confound is often neglected: the mean age (± standard deviation) of the stroke patients included in the above mentioned studies was 57 ± 3.3 years. In contrast, the healthy subjects who participated in the functional imaging studies had a mean age of 29.7 ± 8.4 years. It is well accepted that aging influences cognitive as well as motor processes. The dedifferentiation theory proposes that with aging, cognitive functions increasingly depend on shared neural networks and thus rely less on specialized network nodes (Cabeza, 2001). The attenuated network (in elderly adults) representing a given cognitive function would thus be similar to the original network (in younger adults) in structural terms, but would differ with respect to its functional organization. Alternatively, the compensation account suggests that in the aging brain, additional brain regions are recruited (resulting in structurally altered networks) to secure normal performance (Heuninckx et al., 2008). Studies that examined cognitive aging found support for both theories, and to date the jury is out on this issue. With regard to the motor domain, a recent study by Carp et al. (2011) reported results favoring the dedifferentiation theory. By employing a simple finger tapping task, the authors showed that no additional brain regions were recruited by older subjects compared to younger subjects, but rather the degree of activation changed within these regions, i.e., some of the regions involved showed a greater recruitment in older than in younger subjects.

All lesion locations in (elderly) patients with pantomime deficits affected the left-hemispheric network characterized by functional imaging studies in healthy, young subjects. Therefore, a structurally similar pantomime network was found in both young and elderly subjects. The most parsimonious explanation reconciling the findings of lesion and functional imaging studies therefore implies that according to the dedifferentiation account, specialization of the (inferior) parietal lobe for pantomiming of object use as observed in young subjects may be attenuated in the elderly. This is reflected in the distribution of lesion locations and activated regions when expressed as percentage scores. Of 14 reported lesions (see Fig. 2), 50% were found in the parietal cortex, 22% in the temporal cortex, 14% in the frontal cortex, 7% in the insular cortex and 7% in the fusiform gyrus. On the other hand, of 38 described activation peaks (see Fig. 3), 40% were located in the parietal cortex, 21% in the frontal cortex (with additional 26% in BA6), 8% in the temporal cortex, 2.5% in the occipital gyrus and 2.5% in the insular cortex. This suggests that the prominent role of the parietal cortex for pantomiming is preserved in the aging brain, but that the representation of pantomiming is also distributed in a less focused way as indicated by the differential involvement of frontal and temporal areas in pantomiming in the elderly patients compared to young healthy subjects.

The broader distribution of a given cognitive function in the aging brain proposed by the dedifferentiation theory could lead to a reduced vulnerability of that function to brain lesions. This might seem at odds with our finding that relatively circumscribed lesion sites are associated with pantomime dysfunction in elderly patients. It should be noted, however, that the most frequently affected site within rather large lesions (see Fig. 1a) was used for further analysis in our systematic review. Therefore, we would like to argue that in most cases rather large lesions compromise pantomiming in elderly patients. These rather large lesions can affect even a broadly distributed pantomime representation by disturbing both the critical site for pantomiming (i.e., the parietal lobe) and at the same time (at least some of) the additional sites which became also important parts of the distributed pantomime network in the elderly (i.e., frontal and temporal areas). As current lesion analysis methods are mainly based on lesion frequency, lesion analyses will most likely identify the parietal cortex as the critical site for pantomiming, but will also point to the importance of the other pantomime-related sites within the large lesions causing pantomime dysfunction in the elderly. In fact, that was exactly what we found in our systematic review of the lesion studies of pantomiming object use. Nevertheless, it might also be possible that lesion-induced plasticity and compensatory mechanisms led to differential changes in the neuronal network associated with object-related pantomime in patients and thus contribute to differences in the results of structural lesion versus functional imaging studies.

In order to test this conjecture, future studies could use functional imaging to directly compare the brain activation patterns of young and elderly healthy participants performing pantomimes of object use to elucidate whether the left-hemispheric pantomime network is structurally similar in both age groups. Furthermore, connectivity analyses (for instance dynamic causal modeling, DCM) could help to examine whether these structurally similar pantomime networks show different patterns of connectivity reflecting the changed division of labor within the network of the elderly.

## 5 Conclusions

This review of the current structural lesion and functional imaging studies on the neural basis of pantomiming revealed that pantomime of object use is predominately subserved by a left-hemispheric fronto-parietal network. Within this pantomime network, the parietal cortex plays the key role as indicated by the fact that 11 of 12 functional imaging studies conducted with healthy, young subjects reported parietal activations during pantomiming. More specifically, the (inferior) parietal lobe seems to be crucially involved in the process of activating appropriate motor schemata in the absence of the actual object, one of the core processes of pantomiming. The reviewed ten structural lesion studies in stroke patients also revealed a left-hemispheric fronto-parietal network subserving pantomiming. Therefore, the findings of structural (lesion) and functional imaging studies converge by showing that within the left-hemispheric fronto-parietal pantomime network (inferior) parietal cortex is involved in activating the appropriate motor schemata for pantomiming the use of an object, while the (inferior) frontal cortex subserves their execution. Finally, the comparison of functional imaging studies (in healthy young subjects) with structural lesion studies (in elderly stroke patients) points to a broader distribution of pantomime-related processes in the elderly consistent with the dedifferentiation theory.

## Figures and Tables

**Table 1:** Summary of the structural lesion studies on the neural basis of pantomime.

Author (year)	Patient population (*n*)	Mean age (years)	Task	Hand used	Anatomical description of lesion location
Varney and Damasio (1987)	LBD (100)	57.5	Recognition of pantomimes	n/a	STG; SMG and angular gyrus (both in IPL); fusiform gyrus; BG
Halsband et al. (2001)	LBD (15)	59.1	Recognition of pantomimes	n/a	Parietal cortex
Buxbaum et al. (2003)	LBD (14)	63.5	Execution of pantomimes	Ipsilesional hand	IPL
Buxbaum et al. (2005)	LBD (13)	53.6	Execution of pantomimes	Ipsilesional hand	IPS
Goldenberg et al. (2007)	LBD (44)	53.2	Execution of pantomimes	Ipsilesional hand	IFG
Weiss et al. (2008)	LBD (20)	55.5	Recognition of pantomime	n/a	Angular gyrus (in IPL)
Kalenine et al. (2010)	LBD (43)	56.2	Recognition of pantomime	n/a	IPL, MTG
Dovern et al. (2011)	LBD (43)	53.5	Execution of pantomimes	Ipsilesional hand	n/a
Manuel et al. (2013)	LBD (84)	60.5	Execution of pantomimes	Ipsilesional hand	IFG, MTG
Hermsdörfer et al. (2013)	LBD (23)	57.6	Execution of pantomimes	Ipsilesional hand	insula

Studies are listed in chronological order. BG = basal ganglia; IFG = inferior frontal gyrus; IPL = inferior parietal lobe; IPS = intraparietal sulcus; LBD = patients with left brain damage; MTG = middle temporal gyrus; n/a = not applicable; SMG = supramarginal gyrus; STG = superior temporal gyrus.

**Table 2:** Summary of the functional imaging studies on the neural basis of pantomime.

Study	Subjects' mean age (years)	Method	Experimental paradigm	Modality of presentation	Stimulus	Control condition	MNI coordinates of reported activation peaks
Moll et al. (2000)	30	fMRI	Execution of pantomimes	Auditory/verbal	Object name	Repetition of a multistage sequence of movements performed with forearm, wrist, hand, and fingers	n/a
Choi et al. (2001)	30	fMRI	Execution of pantomimes	Visual	Object name	Oppositional finger movement; rest	SPL (− 36 − 52 58); lateral BA6 (− 16 − 16 69); medial BA6 (− 4 1 70); ITG (− 57 − 57 − 22); cerebellum (− 4 − 89 − 34); putamen (− 16 8 0)
Rumiati et al. (2004)	26.1	PET	Execution of pantomimes	Visual	Picture of the object	Imitation of pantomime	Lateral BA6 (− 30 − 26 70); medial BA6 (− 6 − 17 58); IPL (− 63 − 32 29); insular cortex (− 44 − 2 4); MFG (− 34 33 34); IFG (− 55 5 33); MTG (− 57 − 70 − 2); putamen (− 26 − 4 6)
Ohgami et al. (2004)	29.5	fMRI	Execution of pantomimes	Visual	Object name	Repetitive grasping movement	Medial BA6 (0 8 58); lateral BA6 (− 26 − 2 70); IPL (− 44 − 46 56); SPL (− 30 − 60 66); IFG (− 48 8 28); MFG (− 44 34 26)
Johnson-Frey et al. (2005)		fMRI	Go–nogo pantomime	Auditory/verbal	Object name	Produce random, meaningless movement	IPL (− 36 − 32 42); MTG (− 52 − 64 − 3)
Fridman et al. (2006)	25	fMRI	Go–nogo pantomime w/out object use	Visual	Visually presented verbal command asking for a transitive movement^a^	Visually presented verbal command asking for a intransitive movement*	IPL (− 40 − 51 36); IFG (− 53 8 9); IPS (− 59 − 40 52); SPL (− 22 − 61 64)
Hermsdörfer et al. (2007)	***25.1***	***fMRI***	***Execution of pantomimes***	***Visual***	***Picture of the object***	***Real object use***	***IPS (− 30 − 60 48)***
Imazu et al. (2007)	***26.1***	***fMRI***	***Execution of pantomimes***	***Auditory***	***Not explicitly mentioned***	***Real object use***	***IPL (− 53 − 48 45)***
Kroliczak and Frey (2009)	27	fMRI	Execution of transitive pantomimes	Visual	Verbs denoting to-be pantomimed actions	Execution of intransitive pantomimes	Medial BA6 (− 4 − 8 62)
Bohlhalter et al. (2009)		fMRI	Execution of pantomimes	Visual	Visually presented verbal command asking for a transitive movement^a^	Planning of pantomimes	Ventral BA6 (− 42 − 16 58); medial BA6 (− 8 − 12 52); SPL (− 32 − 50 64)
Vingerhoets et al. (2011)	***33.1***	***fMRI***	***Execution of familiar pantomimes***	***Visual***	***Picture of the object***	***Execution of unfamiliar pantomimes***	***IPL (− 51 − 63 37)***
Vingerhoets et al. (2012)	21.5	fMRI	Unimanual execution of pantomimes	Visual	Picture of the object	Unimanual execution of meaningless movement	MFG (− 33 37 35); IFG (− 53 20 − 6); ventral BA6 (− 46 0 27); SPL (− 31 − 55 66); IPL (− 56 − 22 31); cerebellum (− 34 − 72 − 22); IOG (− 31 − 88 − 5)

Table 2. gives an overview of functional neuroimaging studies that assessed pantomiming behavior in healthy subjects. The modality of presentation indicates how the participants were instructed (either visually by presenting a picture of an object or the object name or verbally by naming the object). Bold and italics print indicates the studies that led to a specific activation of the parietal cortex during pantomiming (without further activation clusters in other brain regions). BA6 = Brodmann Area 6; IFG = inferior frontal gyrus; IPL = inferior parietal lobe; IPS = intraparietal sulcus; IOG = inferior occipital gyrus; ITG = inferior temporal gyrus; MFG = middle frontal gyrus; MTG = middle temporal gyrus; n/a = not applicable; SFG = superior frontal gyrus; SPL = superior parietal lobe.

a In contrast to the studies using the object name (one word) as a stimulus, this study used a full sentence to instruct a transitive/intransitive movement on written verbal command.

**Table 3:** Results of ES-SDM analysis for the functional imaging studies on pantomiming object use (n = 12).

MNI coordinate	z-score	P-value	Number of sig. voxels	Anatomical description
− 40, − 56, 47	3.273	0.000	2570	Left IPL (angular gyrus)
− 59, 5, 20	2.555	0.000	843	Left IFG
− 44, 42, 33	2.003	0.000	314	Left MFG
− 55, − 64, − 8	1.602	0.003	33	Left ITG

The anatomical descriptions of the clusters revealed by the ES-SDM (effect sizes signed differential mapping) approach are obtained by using the Anatomy Toolbox based on MNI-coordinates. All listed clusters are highly significant. IFG = inferior frontal gyrus, IPL = inferior parietal lobe; ITG = inferior temporal gyrus; MFG = middle frontal gyrus.

## Footnotes

1 Note that we prefer to describe the clinical symptomatology of apraxia and refrain from using terms like ideo-motor apraxia or ideational apraxia, as the different apraxia classifications are currently under debate.
